# Transverse Myelitis Associated with Anti-Ro (SSA) Autoantibodies: A Record of Two Cases

**DOI:** 10.1155/2012/515768

**Published:** 2012-11-13

**Authors:** G. Melikyan, M. H. Abdelrahman, A. D'Suoza, N. Akhtar, A. N. Elzouki, M. Hammoudeh

**Affiliations:** ^1^Neurology Section, Department of Medicine, Hamad General Hospital, P.O. Box 3050, Doha, Qatar; ^2^Rheumatology Section, Department of Medicine, Hamad General Hospital, P.O. Box 3050, Doha, Qatar; ^3^General Medicine Section, Department of Medicine, Hamad General Hospital, P.O. Box 3050, Doha, Qatar

## Abstract

Transverse myelitis (TM) is an inflammatory process involving a restricted area of the spinal cord. The usual dramatic presentation makes TM a medical emergency. Early detection and aggressive therapy are required in order to improve the prognosis. The association of this unique clinical phenotype and autoantibody provides circumstantial evidence that an autoimmune aetiology might be involved. We describe two cases of TM associated with anti-Ro (SSA) autoantibodies without connective tissue disease manifestations. The two patients were treated successfully with IV steroids and cyclophosphamide.

## 1. Introduction

Transverse myelitis (TM) is an inflammatory process involving a restricted area of the spinal cord. The usual dramatic presentation makes TM a medical emergency. TM—as a first manifestation—is unusual presentation of some autoimmune diseases like Sjogren's syndrome (SS) and systemic lupus erythematosus (SLE). Typically, TM is monophasic; however, some patients develop recurrent TM without any known associated disease. The association between anti-Ro antibodies and recurrent TM has been reported, which suggests that the mechanism of spinal cord injury may be autoimmune in nature. Early detection and aggressive therapy are required in order to improve the prognosis [1]. 

## 2. Case 1

**Figure 1:**
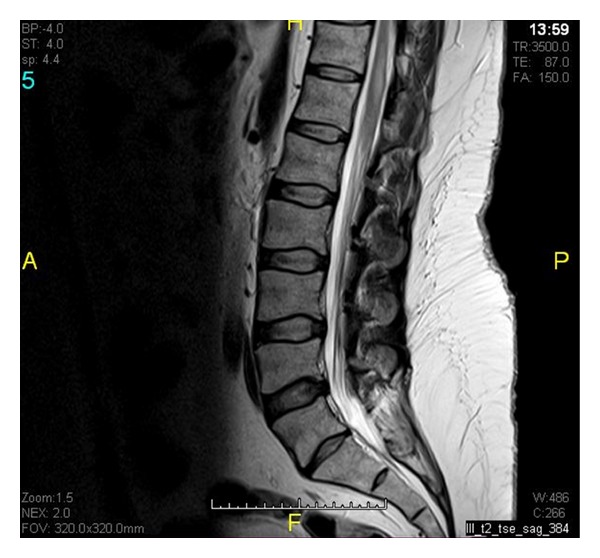
Focal area of high T2 signal change in lower spinal cord from T11-T12 level.

**Figure 2:**
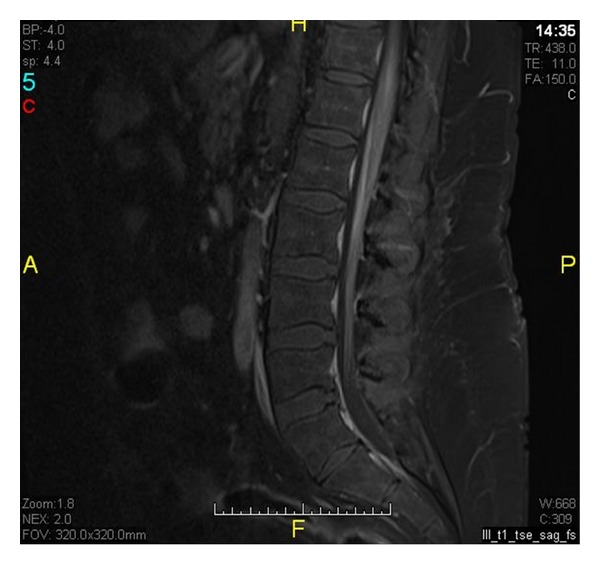
Focal enhancement within posterolateral columns corresponding to the area of high signal changes on T2.

 A 43-year-old male presented with weakness in both legs preceded by tingling sensation, back pain, but no sphincter dysfunction. Two months earlier, he had a similar attack. Neurological examination revealed asymmetric flaccid paraplegia (power 0/5) with areflexia. Funduscopic examination showed no optic neuritis. His investigations showed positive anti-Ro (SSA) autoantibodies. Rheumatoid factor, anti-DNA, anti-Sm, anti-La (SSB), antiribonucleoprotein (RNP), antiphospholipid (APL), and anti-NMO antibodies were negative. Spinal cord Magnetic Resonance Imaging (MRI) showed enhancing hyperintense lesion involving long segment from lower thoracic to conus medullaris confirming the diagnosis of TM on both occasions (Figures 1 and 2). We treated him with intravenous immunoglobulin (IVIG) at a dose of 400 mg/kg/day for five days with significant improvement. On the second admission, the patient received IVIG at the same dose, methylprednisolone at a dose of 1 g/day for five days followed by oral prednisolone at 1 mg/kg, and IV cyclophosphamide at a dose of 500 mg every two weeks. After four weeks of treatment, his power in the lower limbs was 4/5. 

## 3. Case 2

A 29-year-old male presented with a three-day history of fever, low back, and neck pain, followed by rapid bilateral lower limb weakness and retention of urine. His neurologic examination revealed significant neck stiffness and flaccid paraparesis. His investigations showed negative Anti-NMO antibodies, positive antinuclear antibodies (ANA) at 1 : 640, as well as anti-Ro (SSA) and anti-La (SSB) auto-antibodies. Spinal cord MRI showed a hyperintense in T2 and isointense in T1 cord lesion involving all along its length up to lower medulla, with no contrast enhancement confirming the diagnosis of transverse myelitis. Patient treated with IV methyl prednisolone 1 gram for three days and IVIG for five days. Over a period of months, he showed improvement in sphincteric functions and could walk with minimal assistance. 

## 4. Discussion

 Transverse myelitis (TM), a process involving the full thickness of the spinal cord, is a rare event with an estimated incidence of 31 per million people [2]. It is a well-known manifestation of many autoimmune diseases. It was reported to occur in SLE [3], SS [4], antiphospholipid syndrome [5], mixed connective tissue disease [6], scleroderma [7], ankylosing spondylitis [8], Behcet's disease [3], and sarcoidosis [3]. TM may occur in less than 1% of patients with SS and 1–3% of patients with SLE [3]. An association with SS was the most frequent (10%), followed by sarcoidosis (5.9%), SLE (3.8%), and antiphospholipid syndrome (1.4%) [3]. A case-control study [9] at the Johns Hopkins Transverse Myelopathy Center between October 2001 and January 2002 demonstrated thirteen patients with recurrent TM; ten of them found to have anti-Ro (SSA) autoantibodies, and they had 50% response rate to treatment with corticosteroids. The author concluded that the presence of these antibodies might be used as a predictor of recurrence and that they might respond to treatment with immunosuppressive medications, as these antibodies might be a partial expression of connective tissue disease. Treatment with pulse doses of corticosteroids alone may be suboptimal as results of treatment with a combination of corticosteroids and cyclophosphamide have been encouraging [10]. These patients had transverse myelitis (one of them recurrent) without any other manifestations of connective tissue diseases; one of them followed for more than three years. In conclusion, patients with idiopathic transverse myelitis who has autoantibodies like Ro-(SSA) might be at risk for recurrence, which we believe warrants checking for these autoantibodies in all these patients and defend treating them with corticosteroids and immunosuppressive therapy.
